# Second Generation Self-Inflating Tissue Expanders: A Two-Year Experience

**DOI:** 10.1155/2014/457205

**Published:** 2014-01-23

**Authors:** Jamal Omran Al Madani

**Affiliations:** Plastic Surgery Unit, Plastic Surgery Resident, Security Forces Hospital, Riyadh, Saudi Arabia

## Abstract

*Background*. Tissue expansion is a well-established surgical technique that produces an additional amount of normal skin to cover a defect. This technique is appealing because the skin quality and color are from the patient's own. The widely used injectable expanders are of great reliability but carry the disadvantage of being painful during injection and most of the time require multiple clinic visits. So the idea of self-inflation became attractive and hydrogel expanders were developed and became widely known for being painless during clinic visit and decrease number of visits. The first generation expanders were modified by adding an enclosing plastic shell to decrease the unopposed expansion that occurred in the first generation expanders, which lead to pressure necrosis of the skin flaps. This made it an attractive option for tissue expansion in children and some adult patients. *Patients, Materials, and Methods*. Charts of 17 patients were retrospectively reviewed, all of them had second generation self-inflating expanders implanted over a 2-year period for one of two purposes, the treatment of giant nevi or burn scars. *Results*. Fifteen patients were females and 2 were males. The indication was large burn scar in 14 cases (14/17), in which 47/55 expanders were implanted, and giant nevus in 3/17 cases in which 8/55 expanders were implanted. Extrusion of the expander occurred in 8/55 expanders (14.5%), which occurred in 6/14 patients. The highest percentage of extrusion occurred in the neck in which two out of three expanders extruded; otherwise this complication does not seem to be related to the indication, gender, nor age of the patients. It seems to be that it is technical in nature. The patients did not have to get any injections to fill the tissue expanders, which made the expansion process less painful and more convenient. *Conclusion*. This seems to be currently the largest published review in which second generation expanders were used. Those expanders seem to offer a desirable advantage of being painless for children, also they do not require repeated visits to the surgeon's clinic, which is of great value for patients living in the periphery.

## 1. Introduction

Tissue expansion is an important tool in the plastic surgeon's toolbox. First report of this method was in 1957, when Neumann used a rubber balloon to expand skin in the temporooccipital area to reconstruct a traumatized ear [1]. At that time it seemed like a sporadic idea it did not get a lot of publicity. Twenty years later, the idea resurfaced by Radovan and Argenta who used a silicon inflatable expander in breast reconstruction. Afterwards this technique gained a lot of attention [2, 3].

Injectable (conventional) expanders were regularly used for this purpose. The expander needs to be filled manually through an external filling port. This lead to a couple of major disadvantages, the filling process is usually painful when the surgeon introduces the needle into the port underneath the skin and the patient must come to a medical facility regularly in most cases, unless they were trusted with their expanders to be filled by them at their homes.

**Figure 1:**
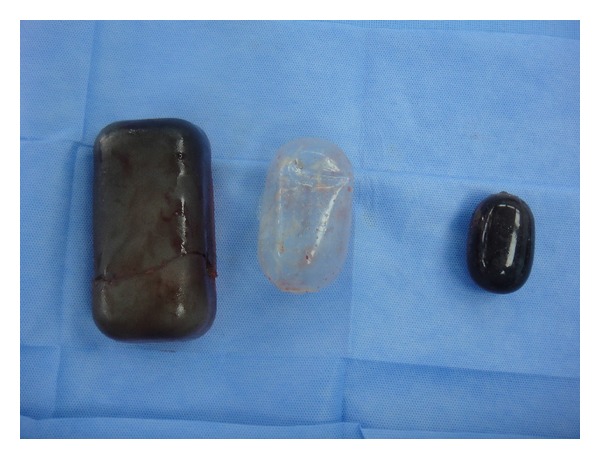
Left expander that has expanded in size beyond the expected size, middle ruptured envelop of the expander on the left, right, expander that expanded to the calculated volume and did not extrude.

In 1982 Austad and Rose developed the first self-inflating tissue expander composed of hypertonic, saturated saline, which was abandoned. The reason was the increased risk of skin necrosis when the fluid leaked from its shell [4]. In 1993, Weise tested a newer hydrogel self-inflating expander on rats, that gave a new horizon for tissue expansion [5]. In 1999 Osmed (Ilmenau, Germany) introduced the self-inflating tissue expanders giving a new option for tissue expansion. The expanders are made from a solid material called hydrogel, vinyl pyrrolidone, and methyl methacrylate material that absorbs the surrounding tissue fluid and increase in size over a period of 6–8 weeks. The company marketing the expander claims that it increases to 10 times the original size, but the increase in size measured in a clinical report on human was 6.9 times the original size in the second generation expanders [6] (see Figure 1).

The first generation expanders rapidly increased in size and extruded outside the body. This caused pressure necrosis over the overlying skin flap. So they were modified by adding an envelope to decrease the unopposed expansion. The envelope is made of silicone and had pores to allow the fluid in. Hence those second generation tissue expanders had a better outcome [7].

This study is a single-center experience, in which 55 rectangular second generation self-inflating tissue expanders were used.

## 2. Materials and Methods

### 2.1. Patients

Seventeen patients had implantation of 55 expanders over a 2-year period, from April, 2010, to April, 2012. All the patients that had tissue expander implanted records were retrospectively reviewed. The data collected included age, gender, indication, site, number of surgeries including insertion and both elective and emergency removal, size of the expanders, and number of days the expander remained in the body. Out of the 17 patients included in the study 15 patients were females and 2 were males. The indication was large burn scar in 14 cases (14/17), in which 47/55 expanders were implanted, and giant nevus in 3/17 cases in which 8/55 expanders were implanted. Three patients were of pediatric age group (less than 14 years) and the remaining were adults. Age ranged from 9 to 40 years and mean age was 19.14 years. The use of those expanders was preferred in pediatrics and in patients living far from Riyadh, the capital of Saudi Arabia, referred from the peripheral hospital in their regions. The aim was to decrease the sessions of painful inflations and the number of visits for patients living away from the hospital. Selection of patients depended on the surgeons' and patients' preference.

### 2.2. Expanders

All expanders were second generation self-inflating tissue expanders produced by Osmed GmbH, Ilmenau, Germany. All used expanders were rectangular in shape. Sizes were 60, 75, 130, 200, and 300 cc predicted final volume as per company box (for details see Table 1).

### 2.3. Course

#### 2.3.1. Implantation

All elective surgeries were performed by a team of two plastic surgery consultants. All were under general anesthesia; preoperatively the normal skin next to the lesion to be removed is marked in a way to create a subcutaneous pocket in which the expander would be inserted. The dimensions of the pocket would be equal to the final dimensions that the expander is supposed to reach as indicated on the company box. This was done in an attempt to make the expander get a bigger room to expand under and to decrease the unopposed pressure on the overlying skin to prevent ischemia and extrusion.

In cases of large lesion that would require the insertion of more than one expander on one side, separate pockets were created to disallow any potential infection to spread from one expander to another in case any infection occurs 2-3 weeks postoperatively. The aim is to keep the uninfected expanders and utilize them later if they did not reach their final predicted volume yet which happens after around 6 weeks.

The surgery would begin by making a 3-4 cm cut inside the lesion territory perpendicular to the expander's pocket; infiltration of around 30–40 cc of tumescent fluid containing epinephrine would follow. Dissection of the pocket using metzenbaum scissors is done leaving a 1 cm thick skin. Atraumatic dissection of the pocket follows. The expander is inserted and the skin is closed using continuous sutures. A course of oral antibiotic for 5 days was given. The implantation process would take around 20 minutes.

#### 2.3.2. Elective Removal

All surgeries were performed under GA. An incision is made in the line between the normal skin and the lesion, the pocket is opened and the expander is removed, and the capsule may be scored if the desired stretch was not achieved. The surface area of the lesion that can be removed is estimated and the skin flaps are advanced; a drain is inserted, which is usually removed after 1-2 days according to the output.

#### 2.3.3. Removal of the Extruded Expanders

When extrusions occurred the patients were admitted through the emergency. Intravenous antibiotics were started at the time of admission, and were taken to the operating room as soon as possible. A similar technique to electively remove the expander is used, but the pocket is thoroughly irrigated and cleaned. In case the expander was inserted together with other expanders the pocket would be inspected to make sure that the infection did not spread to another expander; drains were left in place like other elective cases. In all the cases the infection did not spread and the decision of removing the other expanders electively was made depending on whether the uninfected expander reached a reasonable volume or not. Antibiotics (cefuroxime, dose according to weight) were used regularly to prevent the spread of the infection. Cultures showed staphylococci uniformly. Patients left the hospital after 3–5 days, after the cellulitis has subsided.

## 3. Results

### 3.1. Patients

All patients described were healthy. Only one patient had another illness, which was sickle cell anemia, and no complications occurred during the management of that patient. Two of the patients had the implantation twice due to the very large surface area of the lesion. A minimum of one expander and a maximum of 7 expanders in each patient were implanted. The size of the expanded skin in those self-inflating tissue expanders was similar to the conventional expanders (details of the patients and implanted expanders are in Table 1.)

### 3.2. Complication

All patients reported minimal discomfort and were discharged from the hospital a day after surgery. Extrusion of the expander occurred in 8/55 expanders (14.5%), which occurred in 6/17 patients. Six happened when the indication was burn scar management (75%) and 2 happened when the indication was giant nevus (25%). The extrusion occurred between 30 days and 102 days after implantation with mean of 55.87 days. Out of the eight expanders 2 were inserted in the thigh, 2 in the chest, 1 in the back, 1 in the arm, 2 in the neck, and none in the shoulder or the forearm. Average age in the whole study was 19.14 years; in the group in which the expanders extruded the average was 13.12 years while in the group in which complication did not occur it was 20.19 years. All patients did not have other complications during surgeries and postoperatively until seen in the clinic at least one month later. Neither superficial infection nor ulceration occurred (Details of the patients and the extruded expanders are in Table 2 and rate of complications in relation to site is in Table 3).

## 4. Discussion

After the introduction of the self-inflating tissue expanders many reports were published about the use and versatility of these expanders which included unpphalmia, breast reconstruction, free flap reconstruction, giant nevi and burn scars [6, 7].

The center in which this study was done is a government referral center, in Riyadh, the capital of Saudi Arabia, receiving many patients from smaller peripheral hospitals. Concerning conventional expander to be inserted and the process of expansion to be carried out the surgeon has 2 choices the first is to bring the patient and maybe the family of a child weekly to the clinic. This carries a large number of work and school absences because the clinic time is during the day working hours only. The other choice is to trust the family with the expander and teach them how to inflate it at home, which for many surgeons is not a very reliable option, as it would potentially carry a higher risk of extrusion and infection.

So, this second generation expander came in hand for many patients especially the ones living away from the center and for children. The implantation is usually uncomplicated. Postoperative pain is minimal as the incision is usually small. The number of days in which the expander electively stayed was primarily decided by the social factors for the patients and the availability of operation theater time for the surgeon as well, but it was not less than 42 days and an average (99.1) days.

Extrusion apparently occurs due to pressure necrosis from the unopposed expansion from expander, causing failure of the envelope surrounding the expander. This can be explained by the necrosis of the skin flap seen at the time of removal and debridement.

Extrusion rate for the first generation tissue expanders was as high as 35% in some reports [8, 9], until the second generation that is enclosed by a silicone fenestrated envelop was introduced, after which the complication rate has decreased to some extent. Ronert et al. [7] reported a success rate of 93.3% for tubular breast management, 83.3% for other reconstructions, and 91% for all 26 second generation expanders. While Obdeijn et al. in 2009 reported a lesser success rate which was 70%, they had 20 expanders implanted, but in their experience 2 of the 6 extrusions happened in radiated fields [6]. An impressive 96.2% success rate was reported in 2010 by Böttcher-Haberzeth et al. in a study that was carried on pediatric population in which 53 expanders were implanted; the surgery was short and the expansion process was painless, because the patient did not have to get injections of fluid to fill the expander. No radiation was given to any of the patients but this alone is not the cause of this greater success rate an oversized pocket potentially played an important factor in decreasing the extrusion rate [10].

The results in this study are similar to those reported as the extrusion rate is 14.5%. The extrusion from anatomical areas other than the neck is not remarkable, whereas from the neck 66.6% expanders extruded, which is a limitation in our study, but making this a definitive conclusion is difficult as only 3 expanders were inserted in which 2 extruded. Furthermore in the previous studies [7, 9, 10] no implantations were done in the neck.

Looking at the gender in this study, most of the implantations were carried out in females (52/55) as they would present for cosmetic concerns more than the males who are less concerned about the appearance especially in areas usually covered with clothes. Only 3 expanders were implanted in males. One of those three expanders extruded (33%), and seven out of fifty two (14.3%) in females. There are no previous reports about increased extrusion in either gender with any type of expanders and the number of male patients is very small to make a definite conclusion.

Similarly the indication also does not seem to play a role. Two out of eight expanders extruded in 2 different giant nevi patients but those 2 were in the neck, unlike the other 6 that did not extrude which were implanted in the chest (4/8) and arm (2/8).

In the presented data the pediatrics (less than 14 years) were 6 (in Table 1 their numbers are 3, 4, 7, 12, 13, and 17) and had 27 expanders implanted, 4 of them extruded (14.8%) which is similar to the whole patient group and does not signify an increased risk contrary to the fact that the average age of patients in which the expander extruded was 13.1 years. This number is not as impressive as the one reported before in pediatrics [10].

Two patients had expansion by self-inflating expander twice (patients number 1 and 17 in Table 1). One had an uncomplicated course and the implantation surgeries were around a year apart. The other had 7 expanders implanted and removed without any problems in the first surgery; the patient remained without any expanders for 11 months; then 3 expanders were inserted again in the right thigh and the patient had a complication in the second time with two of the three expanders; the first was removed alone and the second extruded expander when removed was with the last and third expander that did not extrude at all. This is similar to what was reported by Obdeijn et al. [9] in one of their cases where 3 expanders were implanted in a previously expanded area and 1 of them extruded.

Extrusion occurred in a similar rate in the 5 different sizes used (60, 75, 120, 200, and 300 cc). In all the cases the flaps created by the extruded expander were advanced and utilized safely. Extrusion occurred between 29 to 102 days and average 55.8; days the average number of days in which the expander remained without any later complication was 94 days.

At this stage creation of an oversized pocket is advised, similar to the one described by Böttcher-Haberzeth et al. [10]. This was not done in the current study. In this study the pockets dimensions and designs did not account for the vertical height that the expander would reach; only 2 dimensions of the expander were measured and maybe this caused the extrusion.

The disadvantages of this type of expander are that the process is uncontrolled and that the expander cannot be deflated nor reused.

## 5. For Future Studies

The state in which the expanders were found during removal was not documented; in at least one of the cases that was not complicated the expander was outside the enclosing envelop, and in at least one of the cases that were complicated the expander was found in a similar state, which may have caused the extrusion in that patient. But the envelope was intact in some other complicated cases. Important data to be documented is the final size that the expander actually reached and the color the expander got stained with, as some of the expanders were red-color stained which may mean that the blood altered the osmotic inflation process, which are all were lacking in our study and psychological trauma. There is some inconsistency in the reports with regard to the final volume reached in vivo between 10 times and 6.5 times [8, 9, 11]. In a study about hydrogel expansion Weise et al. addressed different inflation behavior in different media in vitro only and responses to blood and serous fluid were not tested [12], which places the final volume proposed by the company on its boxes in doubt. Cost effectiveness cannot be determined in this center because it is a government facility and care including the expanders, nurse clinic, and operating theater time is for free. Nonfinancial benefit in terms of absence of pain are psychological trauma and missing school classes weekly for inflating the expander maybe evaluated in a comparative study.

## 6. Conclusion

(1) Second generation tissue expanders look very attractive for burn scar and giant nevi reconstruction. (2) The neck maybe an area for a high extrusion risk, but this needs to be looked at after more cases are done in the neck. Other anatomical areas like age, gender, size of the expander, and indication do not seem to play a role in extrusion. (3) An oversized pocket is advised. (4) The expander can stay for around 100 days without extrusion, but removal as soon as the expander reaches the required size is advised. (5) If extrusion occurs the expanded skin can still be utilized. (6) Reexpansion seems safe but should be further studied and evaluated.

## Figures and Tables

**Table 1:** Patients and expanders data.

Patient order	Age in years	Gender	Other illnesses	Indication	Order of implantation surgery	Final size of the expander (cc)	Duration (days)	Location	Extruded
1	38	Female	Sickle cell anemia	Burn scar	1st	130	47	Arm	No
						130	47	Arm	No
					2nd	130	100	Arm	No
						75	100	Arm	No

2	21	Female	None	Burn scar	1st	75	102	Chest	Yes
						60	171	Chest	No
						60	171	Chest	No

3	10	Male	None	Nevus	1st	60	52	Neck	Yes

4	9	Female	None	Nevus	1st	60	42	Chest	No
						60	42	Chest	No
						130	42	Neck	Yes

5	29	Female	None	Burn scar	1st	75	126	Forearm	No
						75	126	Forearm	No

6	25	Female	None	Burn scar	1st	75	69	Neck	No

7	11	Female	None	Burn scar	1st	130	63	Chest	No
						200	63	Chest	No

8	18	Female	None	Burn scar	1st	200	46	Shoulder	No

9	38	Female	None	Burn scar	1st	75	167	Arm	No
						130	167	Arm	No

10	40	Male	None	Burn scar	1st	200	99	Forearm	No
						200	99	Forearm	No

11	28	Female	None	Burn scar	1st	300	165	Shoulder	No
						200	165	Shoulder	No
						130	165	Shoulder	No
						130	165	Shoulder	No

12	11	Female	None	Nevus	1st	75	102	Chest	No
						130	102	Chest	No
						60	102	Arm	No
						60	102	Arm	No

13	11	Female	None	Burn scar	1st	60	112	Chest	No
						60	112	Chest	No
						60	112	Chest	No
						75	112	Chest	No
						75	112	Chest	No
						130	112	Chest	No
						300	30	Arm	Yes

14	33	Female	None	Burn scar	1st	60	105	Shoulder	No

15	21	Female	None	Burn scar	1st	75	86	Forearm	No
						75	86	Forearm	No

16	15	Female	None	Burn scar	1st	75	85	Back	No
						300	85	Back	No
						130	85	Back	No
						130	55	Back	Yes
						200	85	Back	Yes

17	12	Female	None	Burn scar	1st	130	120	Thigh	No
						130	120	Thigh	No
						200	120	Thigh	No
						200	120	Thigh	No
						75	120	Thigh	No
						75	120	Thigh	No
						300	120	Thigh	No
						300	29	Thigh	Yes
	13				2nd	300	52	Thigh	No
						60	52	Thigh	Yes

**Table 2:** Patients and expanders of complicated cases.

Duration (days)	Size (cc)	Age	Gender	Indication	Location	Extruded	Other illnesses
102	75	21	Female	Burn scar	Chest	Yes	None
52	60	10	Male	Nevus	Neck	Yes	None
42	130	9	Female	Nevus	Neck	Yes	None
30	300	11	Female	Burn scar	Arm	Yes	None
55	130	15	Female	Burn scar	Back	Yes	None
85	200	15	Female	Burn scar	Back	Yes	None
29	300	12	Female	Burn scar	Thigh	Yes	None
52	65	12	Female	Burn scar	Thigh	Yes	None

**Table 3:** Rate of complications in relation to site.

Location	Count	%
Arm	1	12.5
Forearm	0	0
Chest	1	12.5
Neck	2	25
Shoulder	0	0
Back	2	25
Thigh	2	25

## References

[1] Neumann CG (1957). The expansion of an area of skin by progressive distension of a subcutaneous balloon. *Plastic and Reconstructive Surgery*.

[2] Radovan C (1979). Development of adjacent flaps using a temporary expander. *Plastic Surgery Forum*.

[3] Argenta LC (1984). Reconstruction of the breast by tissue-expansion. *Clinics in Plastic Surgery*.

[4] Austad ED, Rose GL A self-inflating tissue expander. *Plastic and Reconstructive Surgery*.

[5] Wiese KG (1993). Osmotically induced tissue expansion with hydrogels: a new dimension in tissue expansion? A preliminary report. *Journal of Cranio-Maxillo-Facial Surgery*.

[6] Obdeijn MC, Nicolai JPA, Werker PMN (2009). The osmotic tissue expander: a three-year clinical experience. *Journal of Plastic, Reconstructive and Aesthetic Surgery*.

[7] Ronert MA, Hofheinz H, Manassa E, Asgarouladi H, Olbrisch RR (2004). The beginning of a new era in tissue expansion: self-filling osmotic tissue expander—four-year clinical experience. *Plastic and Reconstructive Surgery*.

[8] Chummun S, Addison P, Stewart KJ (2010). The osmotic tissue expander: a 5-year experience. *Journal of Plastic, Reconstructive and Aesthetic Surgery*.

[9] Obdeijn MC, Nicolai JPA, Werker PMN (2009). The osmotic tissue expander: a three-year clinical experience. *Journal of Plastic, Reconstructive and Aesthetic Surgery*.

[10] Böttcher-Haberzeth S, Kapoor S, Meuli M (2011). Osmotic expanders in children: no filling—no control—no problem?. *European Journal of Pediatric Surgery*.

[11] Lohana P, Moiemen NS, Wilson YT (2012). The use of Osmed (TM) tissue expanders in paediatric burns reconstruction. *Annals of Burns and Fire Disasters*.

[12] Weise KG, Heinemann DE, Ostermeier D, Peters JH (2001). Biomaterial properties and biocompatibility in cell culture of a novel self-inflating hydrogel tissue expander. *Journal of Biomedical Materials Research*.

